# Effect of neighborhood deprivation index on breast cancer survival in the United States

**DOI:** 10.1007/s10549-023-07053-4

**Published:** 2023-08-05

**Authors:** Arya Mariam Roy, Anthony George, Kristopher Attwood, Sabah Alaklabi, Archit Patel, Angela R. Omilian, Song Yao, Shipra Gandhi

**Affiliations:** 1 Division of Hematology and Oncology, Department of Medicine, Roswell Park Comprehensive Cancer Center, Buffalo, NY 14263 USA; 2 Department of Biostatistics, Roswell Park Comprehensive Cancer Center, Buffalo, NY 14228 USA; 3 Division of Oncology, Department of Medicine, King Faisal Specialist Hospital and Research Center, Riyadh, Saudi Arabia; 4 Department of Cancer Prevention and Control, Roswell Park Comprehensive Cancer Center, Buffalo, NY 14228 USA

**Keywords:** Socioeconomic status, Neighborhood deprivation, Breast cancer, Health care disparities, Locoregional breast cancer, Overall survival

## Abstract

**Purpose:**

To analyze the association between the Neighborhood Deprivation Index (NDI) and clinical outcomes of locoregional breast cancer (BC).

**Methods:**

Surveillance, Epidemiology and End Results (SEER) database is queried to evaluate overall survival (OS) and disease-specific survival (DSS) of early- stage BC patients diagnosed between 2010 and 2016. Cox multivariate regression was performed to measure the association between NDI (Quintiles corresponding to most deprivation (Q1), above average deprivation (Q2), average deprivation (Q3), below average deprivation (Q4), least deprivation (Q5)) and OS/DSS.

**Results:**

Of the 88,572 locoregional BC patients, 27.4% (n = 24,307) were in the Q1 quintile, 26.5% (n = 23,447) were in the Q3 quintile, 17% (n = 15,035) were in the Q2 quintile, 13.5% (n = 11,945) were in the Q4 quintile, and 15.6% (n = 13,838) were in the Q5 quintile. There was a predominance of racial minorities in the Q1 and Q2 quintiles with Black women being 13–15% and Hispanic women being 15% compared to only 8% Black women and 6% Hispanic women in the Q5 quintile (p < 0.001). In multivariate analysis, in the overall cohort, those who live in Q2 and Q1 quintile have inferior OS and DSS compared to those who live in Q5 quintile (OS:- Q2: Hazard Ratio (HR) 1.28, Q1: HR 1.2; DSS:- Q2: HR 1.33, Q1: HR 1.25, all p < 0.001).

**Conclusion:**

Locoregional BC patients from areas with worse NDI have poor OS and DSS. Investments to improve the socioeconomic status of areas with high deprivation may help to reduce healthcare disparities and improve breast cancer outcomes.

## Introduction

Breast cancer (BC) is the most common malignancy in women globally and the most common cause of cancer-associated mortality [1]. Several demographics, clinicopathological factors including size, grade of the tumor, lymph node (LN) status, hormone receptor status, metastasis, age, and comorbidities of the patients are known to be associated with BC survival [2–4]. In addition to this, racial and ethnic backgrounds are also associated with breast cancer survival; Black women have 40% higher age-adjusted BC mortality than non-Hispanic White women [3, 5]. The socioeconomic status (SES) of an individual and the neighborhood consists of multiple variables including income, education, occupation, living conditions which have been reported in relation to the survival of various cancers [6–8]. Neighborhood deprivation index (NDI) is a validated statistical measurement tool to assess the level of disadvantage within the specific neighborhood. It provides a quantitative measure of socio-economic status of community based on various indicators such as education, employment, income, living condition and basic access to services [9].

Reducing racial and ethnic and socioeconomic disparities in the access to health care have long been a major health policy goal in the United States (US). SES of the individual and neighborhood plays an important role in patient’s access to the health system [10]. Owing to the inequalities in opportunities, education, income, and developmental infrastructures, the areas with underprivileged individual and neighborhood SES may be associated with poor prognosis of certain malignancies and worse outcomes through multiple pathways [10, 11]. Patients with low individual SES may not be able to adhere to cancer screening guidelines, which may occur due to their lack of awareness of diseases or access to screening/prevention methods, lack of insurance or other cost-barriers, and/or mistrust of physicians/health sector [12, 13]. In addition to this, socioeconomically underprivileged neighborhoods lack comprehensive healthcare resources, established referral systems, adequate social support, resources to promote healthy lifestyle, and adequate transportation system to access to healthcare from the diagnosis to survivorship [11, 14, 15]. The myriad events rooting from low SES affect cancer-related mortality and morbidity in vulnerable populations.

Although there were some studies done in the past evaluating the association of individual SES with the survival of cancer, epidemiological studies aiming at the association of SES and geographical variation in BC outcomes are limited. Given the fact that most of the factors owing to low socioeconomic conditions are modifiable, it is very relevant to understand them and develop strategies to mitigate health disparities which aid us in improving health-related outcomes. In our study, we examine the association of neighborhood deprivation with the BC-related outcomes in patients with locoregional BC in the US.

## Methodology

### Data sources

#### Neighborhood deprivation index

In our analysis, we used the NDI which encompasses various factors such as wealth and income, education, occupation, and housing conditions. The NDI for each census tract in the US was created using factor analysis, which identified key variables from 13 measures from the above dimensions proposed by Roux and Mair in their study assessing the contribution of neighborhood or residential environments to social and ethnic inequalities in health [16].

“The key variables that are used from wealth and income are median household income, percent of household receiving dividends interest or rental income, percent of households receiving public assistance, median home value, percent of families with incomes below the poverty level. The variables from other dimensions are as follows: education (percent with a high school degree or higher; percent with a college degree or higher), occupation (percent in a management, business, science, or arts occupation; percent unemployed), and housing conditions (percent of households that are female-headed with any children under 18; percent of housing units that are owner occupied; percent of households without a telephone; percent of households without complete plumbing facilities) [9]. NDI values range from -3.6 to + 2.8 and higher values indicate more neighborhood deprivation which implies lower socioeconomic status”. We used the NDI quintiles weighted by the tract population for the analysis. The first NDI quintile corresponds to most deprivation (Q1), second quintile (above average deprivation- Q2), third quintile (average deprivation- Q3), fourth quintile (below average deprivation (Q4)) and fifth quintile corresponds to least deprivation (Q5) [17].

#### Patient selection

**Fig. 1:**
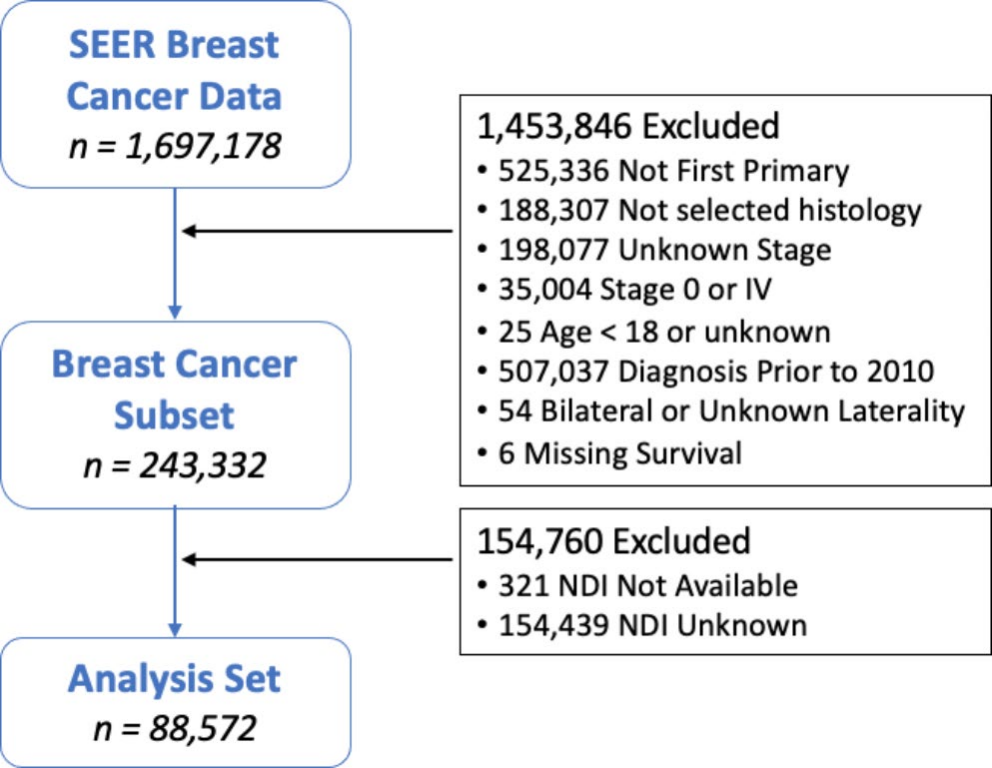
Flow diagram of patient selection schema SEER: Surveillance, Epidemiology, and End Results, n: number, NDI: Neighborhood deprivation index

We queried the Surveillance, Epidemiology and End Results (SEER) registry November 2021 submission database which covers approximately 48% of the US population for our study. We included locoregional BC pts (clinical stage group I, II, III), aged > = 18 years, who were diagnosed from 2010 to 2016, and studied the overall survival (OS) and disease-specific survival (DSS) of BC in association with NDI. Patients were selected from 2010 to 2016 which allowed inclusion of patients with accurate HER2-neu status as accurately captured in SEER from 2010 onwards and adequate 5 years follow up. We excluded patients with unknown or missing data for each variable studied, or clinical/pathological evidence of distant metastases at the time of initial diagnosis. The flow diagram depicting patient selection is shown in Fig. 1. Institutional review board review was exempted as the data were deidentified and from publicly available databases upon request.

### Statistical analysis

The demographical and clinical characteristics of patients by NDI were tabulated by summary statistics. The mean, median, standard deviation, and range were used for continuous variables and the Kruskal-Wallis test was used for comparisons. For the categorical variables, frequencies and relative frequencies were compared using the chi-square test. The median, 3-year, 5-year OS and DSS were summarized by NDI using standard Kaplan-Meier methods.

Cox multivariate regression modeling was performed to test the association between NDI and OS, DSS, with adjustment for age, race, stage, grade, insurance status, surgery, radiation, and chemotherapy (CT). Subset analysis was done based on the BC subtypes (Estrogen receptor and/or progesterone receptor positive HER2-neu negative (HR+), HER2-neu-positive (HER2+), and triple-negative breast cancer (TNBC). All statistics were performed using SAS software version 9.4 (SAS Institute Inc.) and significance testing was 2-sided at p < 0.05. Data were analyzed from June 1, 2022 through July 15, 2022.

## Results

### Patient demographics

The baseline characteristics of the overall cohort are shown in Table 1. Of the 88,572 locoregional BC patients, 27.4% (n = 24,307) were in the most deprivation (Q1) quintile, 17% (n = 15,035) were in the above average deprivation (Q2) quintile, 26.5% (n = 23,447) were in the average deprivation (Q3) quintile, 13.5% (n = 11,945) were in the below average deprivation (Q4) quintile, and 15.6% (n = 13,838) were in the least deprivation (Q5) quintile. The median age of patients in the Q5 quintile was 59 years and Q1 quintile was 61 years (p < 0.001). There was a predominance of racial (p < 0.001) and ethnic (p < 0.001) minorities in the most deprived (Q1) quintile (12.9% Black, 14.8% Hispanic) compared to least deprived (Q5) quintile (8.2% Black, 6.0% Hispanic) (Table 1). There was a higher percentage of uninsured patients in the Q1 quintile compared to the Q5 quintile (2.2% vs. 1.7%, p < 0.001). There were more rural areas in Q1 quintile compared to Q5 quintile (25.9% vs. only 0.7%, p < 0.001). Patients with stage III and grade III disease were observed to be higher in Q1 quintile compared to Q5 quintile (Stage III: 28.7% vs. 14.2%, Grade III: 34% vs. 31.9%, p < 0.001) and a greater percentage of patients received CT in Q1 quintile compared to Q5 quintile (44.6% vs. 42.1%, p < 0.001). However, fewer patients underwent surgery and radiation in the Q1 compared to the Q5 quintile, with 96.1% and 49.7% of patients undergoing surgery and radiation in Q1 quintile compared to 97.1% and 56.5% in the Q5 quintile (p < 0.001 for both, Table 1). There was a higher percentage of aggressive cancers such as TNBC and HER2 + BC in Q1 quintile compared to Q5 quintile (14.5%, 17.7% vs. 11.7%, 16.5% respectively, p < 0.001). The baseline characteristics were stratified by the subtype of breast cancer as shown in Tables 2, 3 and 4. It was observed that the patterns are similar in all the subtypes as observed in the overall cohort except that the patients who received chemotherapy for the locoregional BC were higher in the Q5 when compared to the Q1 in both TNBC and HER2 + BCs.

**Table 1:** Baseline characteristics by NDI in the locoregional breast cancer overall cohort

		Least Deprivation (Q5)	Below Avg Deprivation (Q4)	Average Deprivation (Q3)	Above Avg Deprivation (Q2)	Most Deprivation (Q1)	P-value
	N	13,838 (15.6)	11,945 (13.5)	23,447 (26.5)	15,035 (17.0)	24,307 (27.4)	
Age	Mean/Std/N	59.77/13.33/13,838	60.55/13.33/11,945	60.57/13.24/23,447	60.97/13.16/15,035	60.57/13.18/24,307	< 0.001
	Median/Min/Max	59.00/20.00/102.00	60.00/19.00/100.00	61.00/17.00/102.00	61.00/19.00/108.00	61.00/20.00/102.00	
Sex	Female	13,731 (99.2%)	11,866 (99.3%)	23,265 (99.2%)	14,924 (99.3%)	24,156 (99.4%)	0.238
	Male	107 (0.8%)	79 (0.7%)	182 (0.8%)	111 (0.7%)	151 (0.6%)	
Race	White	11,628 (84.0%)	10,260 (85.9%)	19,746 (84.2%)	12,315 (81.9%)	20,124 (82.8%)	< 0.001
	Black	1,136 (8.2%)	850 (7.1%)	2,657 (11.3%)	2,293 (15.3%)	3,147 (12.9%)	
	Other	1,074 (7.8%)	835 (7.0%)	1,044 (4.5%)	427 (2.8%)	1,036 (4.3%)	
Hispanic	No	13,013 (94.0%)	10,726 (89.8%)	21,892 (93.4%)	14,276 (95.0%)	20,718 (85.2%)	< 0.001
	Yes	825 (6.0%)	1,219 (10.2%)	1,555 (6.6%)	759 (5.0%)	3,589 (14.8%)	
Insurance	Insured	13,597 (98.3%)	11,693 (97.9%)	23,064 (98.4%)	14,725 (97.9%)	23,769 (97.8%)	< 0.001
	Uninsured	241 (1.7%)	252 (2.1%)	383 (1.6%)	310 (2.1%)	538 (2.2%)	
Location	Urban	13,736 (99.3%)	10,395 (87.0%)	17,517 (74.7%)	8,375 (55.7%)	18,012 (74.1%)	< 0.001
	Rural	102 (0.7%)	1,550 (13.0%)	5,930 (25.3%)	6,660 (44.3%)	6,295 (25.9%)	
Grade	I/II	8,962 (64.8%)	7,585 (63.5%)	14,914 (63.6%)	9,318 (62.0%)	15,091 (62.1%)	< 0.001
	III	4,409 (31.9%)	3,791 (31.7%)	7,728 (33.0%)	5,117 (34.0%)	8,271 (34.0%)	
	Unknown	467 (3.4%)	569 (4.8%)	805 (3.4%)	600 (4.0%)	945 (3.9%)	
Stage	I/II	12,266 (15.8)	10,507 (13.6)	20,608 (26.6)	12,968 (16.7)	21,122 (27.3)	< 0.001
	III	1,572 (14.2)	1,438 (13.0)	2,839 (25.6)	2,067 (18.6)	3,185 (28.7)	
Lateral	Left	6,941 (50.2%)	6,025 (50.4%)	11,940 (50.9%)	7,747 (51.5%)	12,343 (50.8%)	0.183
	Right	6,897 (49.8%)	5,920 (49.6%)	11,507 (49.1%)	7,288 (48.5%)	11,964 (49.2%)	
Surgery	Yes	13,439 (97.1%)	11,440 (95.8%)	22,601 (96.4%)	14,501 (96.4%)	23,358 (96.1%)	< 0.001
	No	393 (2.8%)	496 (4.2%)	831 (3.5%)	504 (3.4%)	904 (3.7%)	
	Unknown	6 (0.0%)	9 (0.1%)	15 (0.1%)	30 (0.2%)	45 (0.2%)	
Radiation	Yes	7,821 (56.5%)	6,373 (53.4%)	12,731 (54.3%)	8,019 (53.3%)	12,076 (49.7%)	< 0.001
	No	5,971 (43.1%)	5,495 (46.0%)	10,603 (45.2%)	6,908 (45.9%)	12,121 (49.9%)	
	Total	46 (0.3%)	77 (0.6%)	113 (0.5%)	108 (0.7%)	110 (0.5%)	
Chemo therapy	Yes	5,825 (42.1%)	4,962 (41.5%)	9,970 (42.5%)	6,750 (44.9%)	10,853 (44.6%)	< 0.001
	None/Unknown	8,013 (57.9%)	6,983 (58.5%)	13,477 (57.5%)	8,285 (55.1%)	13,454 (55.4%)	
Subtype	Triple negative	1,390 (11.7%)	1,280 (12.7%)	2,653 (13.3%)	1,843 (14.5%)	2,960 (14.5%)	< 0.001
	h+	8,511 (71.8%)	7,113 (70.6%)	13,994 (70.3%)	8,640 (68.0%)	13,850 (67.8%)	
	HER2+	1,956 (16.5%)	1,689 (16.8%)	3,264 (16.4%)	2,225 (17.5%)	3,625 (17.7%)	

**Table 2:** Baseline characteristics by NDI in locoregional triple negative breast cancer subtypes

		Least Deprivation (Q5)	Below Avg Deprivation (Q4)	Average Deprivation (Q3)	Above Avg Deprivation (Q2)	Most Deprivation (Q1)	P-value
	N	1,390 (13.7)	1,280 (12.6)	2,653 (26.2)	1,843 (18.2)	2,960 (29.2)	
Age	Mean/Std/N	56.76/13.52/1390	57.92/13.82/1280	57.98/13.73/2653	57.26/13.62/1843	57.36/13.70/2960	0.055
	Median/Min/Max	56.00/24.00/102.00	57.00/24.00/96.00	58.00/22.00/100.00	57.00/24.00/99.00	57.00/24.00/96.00	
Sex	Female	1,389 (99.9%)	1,280 (100.0%)	2,651 (99.9%)	1,839 (99.8%)	2,957 (99.9%)	0.389
	Male	1 (0.1%)	0	2 (0.1%)	4 (0.2%)	3 (0.1%)	
Race	White	1,069 (76.9%)	1,006 (78.6%)	2,031 (76.6%)	1,320 (71.6%)	2,198 (74.3%)	< 0.001
	Black	216 (15.5%)	186 (14.5%)	502 (18.9%)	483 (26.2%)	670 (22.6%)	
	Other	105 (7.6%)	88 (6.9%)	120 (4.5%)	40 (2.2%)	92 (3.1%)	
Hispanic	No	1,285 (92.4%)	1,125 (87.9%)	2,445 (92.2%)	1,742 (94.5%)	2,501 (84.5%)	< 0.001
	Yes	105 (7.6%)	155 (12.1%)	208 (7.8%)	101 (5.5%)	459 (15.5%)	
Insurance	Insured	1,354 (97.4%)	1,252 (97.8%)	2,590 (97.6%)	1,785 (96.9%)	2,865 (96.8%)	0.170
	Uninsured	36 (2.6%)	28 (2.2%)	63 (2.4%)	58 (3.1%)	95 (3.2%)	
Location	Urban	1,379 (99.2%)	1,126 (88.0%)	2,037 (76.8%)	1,023 (55.5%)	2,163 (73.1%)	< 0.001
	Rural	11 (0.8%)	154 (12.0%)	616 (23.2%)	820 (44.5%)	797 (26.9%)	
Grade	I/II	225 (16.2%)	221 (17.3%)	450 (17.0%)	331 (18.0%)	520 (17.6%)	0.091
	III	1,135 (81.7%)	1,011 (79.0%)	2,127 (80.2%)	1,446 (78.5%)	2,328 (78.6%)	
	Unknown	30 (2.2%)	48 (3.8%)	76 (2.9%)	66 (3.6%)	112 (3.8%)	
Stage	I/II	1,184 (14.1%)	1,080 (12.8%)	2,211 (26.3%)	1,511 (17.9%)	2,436 (28.9%)	0.069
	III	206 (12.1%)	200 (11.7%)	442 (25.9%)	332 (19.5%)	524 (30.8%)	
Lateral	Left	681 (49.0%)	655 (51.2%)	1,343 (50.6%)	949 (51.5%)	1,521 (51.4%)	0.615
	Right	709 (51.0%)	625 (48.8%)	1,310 (49.4%)	894 (48.5%)	1,439 (48.6%)	
Surgery	Yes	1,340 (96.4%)	1,208 (94.4%)	2,534 (95.5%)	1,779 (96.5%)	2,822 (95.3%)	0.003
	No	50 (3.6%)	71 (5.5%)	114 (4.3%)	56 (3.0%)	133 (4.5%)	
	Unknown	0	1 (0.1%)	5 (0.2%)	8 (0.4%)	5 (0.2%)	
Radiation	Yes	737 (53.0%)	652 (50.9%)	1,369 (51.6%)	1,011 (54.9%)	1,482 (50.1%)	0.093
	No	649 (46.7%)	623 (48.7%)	1,271 (47.9%)	823 (44.7%)	1,468 (49.6%)	
	Total	4 (0.3%)	5 (0.4%)	13 (0.5%)	9 (0.5%)	10 (0.3%)	
Chemotherapy	Yes	1,087 (78.2%)	945 (73.8%)	2,042 (77.0%)	1,425 (77.3%)	2,293 (77.5%)	0.065
	None/Unknown	303 (21.8%)	335 (26.2%)	611 (23.0%)	418 (22.7%)	667 (22.5%)	
Subtype	Triple negative	1,390 (100.0%)	1,280 (100.0%)	2,653 (100.0%)	1,843 (100.0%)	2,960 (100.0%)	< 0.001

**Table 3:** Baseline characteristics by NDI in locoregional hormone receptor positive (HR+) subtype

		Least Deprivation (Q5)	Below Avg Deprivation (Q4)	Average Deprivation (Q3)	Above Avg Deprivation (Q2)	Most Deprivation (Q1)	P-value
1	N	8,511 (16.3)	7,113 (13.7)	13,994 (26.9)	8,640 (16.6)	13,850 (26.6)	
Age	Mean/Std/N	60.80/13.01/8511	61.37/13.02/7113	61.46/12.91/13,994	61.89/12.73/8640	61.64/12.84/13,850	< 0.001
	Median/Min/Max	61.00/24.00/101.00	62.00/19.00/100.00	62.00/22.00/97.00	62.00/19.00/100.00	62.00/21.00/102.00	
Sex	Female	8,438 (99.1%)	7,060 (99.3%)	13,867 (99.1%)	8,567 (99.2%)	13,736 (99.2%)	0.809
	Male	73 (0.9%)	53 (0.7%)	127 (0.9%)	73 (0.8%)	114 (0.8%)	
Race	White	7,339 (86.2%)	6,246 (87.8%)	12,178 (87.0%)	7,309 (84.6%)	11,888 (85.8%)	< 0.001
	Black	541 (6.4%)	381 (5.4%)	1,229 (8.8%)	1,079 (12.5%)	1,399 (10.1%)	
	Other	631 (7.4%)	486 (6.8%)	587 (4.2%)	252 (2.9%)	563 (4.1%)	
Hispanic	No	8,046 (94.5%)	6,449 (90.7%)	13,123 (93.8%)	8,231 (95.3%)	11,880 (85.8%)	< 0.001
	Yes	465 (5.5%)	664 (9.3%)	871 (6.2%)	409 (4.7%)	1,970 (14.2%)	
Insurance	Insured	8,385 (98.5%)	6,962 (97.9%)	13,822 (98.8%)	8,496 (98.3%)	13,599 (98.2%)	< 0.001
	Uninsured	126 (1.5%)	151 (2.1%)	172 (1.2%)	144 (1.7%)	251 (1.8%)	
Location	Urban	8,454 (99.3%)	6,177 (86.8%)	10,381 (74.2%)	4,873 (56.4%)	10,340 (74.7%)	< 0.001
	Rural	57 (0.7%)	936 (13.2%)	3,613 (25.8%)	3,767 (43.6%)	3,510 (25.3%)	
Grade	I/II	6,814 (80.1%)	5,537 (77.8%)	11,177 (79.9%)	6,786 (78.5%)	10,840 (78.3%)	< 0.001
	III	1,491 (17.5%)	1,279 (18.0%)	2,468 (17.6%)	1,582 (18.3%)	2,591 (18.7%)	
	Unknown	206 (2.4%)	297 (4.2%)	349 (2.5%)	272 (3.1%)	419 (3.0%)	
Stage	I/II	7,724 (16.5%)	6,415 (13.7%)	12,626 (26.9%)	7,689 (16.4%)	12,443 (26.5%)	0.0024
	III	787 (15.1%)	698 (13.4)	1,368 (26.3%)	951 (18.3%)	1,407 (27.0)	
Lateral	Left	4,293 (50.4%)	3,552 (49.9%)	7,075 (50.6%)	4,410 (51.0%)	7,002 (50.6%)	0.747
	Right	4,218 (49.6%)	3,561 (50.1%)	6,919 (49.4%)	4,230 (49.0%)	6,848 (49.4%)	
Surgery	Yes	8,317 (97.7%)	6,885 (96.8%)	13,605 (97.2%)	8,396 (97.2%)	13,439 (97.0%)	< 0.001
	No	190 (2.2%)	222 (3.1%)	384 (2.7%)	236 (2.7%)	389 (2.8%)	
	Unknown	4 (0.0%)	6 (0.1%)	5 (0.0%)	8 (0.1%)	22 (0.2%)	
Radiation	Yes	5,020 (59.0%)	4,018 (56.5%)	7,961 (56.9%)	4,786 (55.4%)	7,234 (52.2%)	< 0.001
	No	3,460 (40.7%)	3,040 (42.7%)	5,955 (42.6%)	3,787 (43.8%)	6,555 (47.3%)	
	Total	31 (0.4%)	55 (0.8%)	78 (0.6%)	67 (0.8%)	61 (0.4%)	
Chemotherapy	Yes	2,494 (29.3%)	2,064 (29.0%)	4,023 (28.7%)	2,710 (31.4%)	4,277 (30.9%)	< 0.001
	None/Unknown	6,017 (70.7%)	5,049 (71.0%)	9,971 (71.3%)	5,930 (68.6%)	9,573 (69.1%)	
Subtype	HR+	8,511 (100.0%)	7,113 (100.0%)	13,994 (100.0%)	8,640 (100.0%)	13,850 (100.0%)	

**Table 4:** Baseline characteristics by NDI in locoregional HER2-positive (HER2+) subtype

		Least Deprivation (Q5)	Below Avg Deprivation (Q4)	Average Deprivation (Q3)	Above Avg Deprivation (Q2)	Most Deprivation (Q1)	P-value
	N	1,956 (15.3)	1,689 (13.2)	3,264 (25.6)	2,225 (17.4)	3,625 (28.4)	
Age	Mean/Std/N	55.62/13.16/1956	56.91/13.38/1689	57.32/13.42/3264	58.74/13.39/2225	57.76/13.15/3625	< 0.001
	Median/Min/Max	54.00/20.00/96.00	56.00/20.00/96.00	57.00/17.00/102.00	58.00/24.00/94.00	57.00/20.00/100.00	
Sex	Female	1,941 (99.2%)	1,679 (99.4%)	3,233 (99.1%)	2,212 (99.4%)	3,615 (99.7%)	0.009
	Male	15 (0.8%)	10 (0.6%)	31 (0.9%)	13 (0.6%)	10 (0.3%)	
Race	White	1,591 (81.3%)	1,403 (83.1%)	2,633 (80.7%)	1,777 (79.9%)	2,890 (79.7%)	< 0.001
	Black	175 (8.9%)	138 (8.2%)	444 (13.6%)	375 (16.9%)	548 (15.1%)	
	Other	190 (9.7%)	148 (8.8%)	187 (5.7%)	73 (3.3%)	187 (5.2%)	
Hispanic	No	1,821 (93.1%)	1,490 (88.2%)	3,005 (92.1%)	2,101 (94.4%)	3,023 (83.4%)	< 0.001
	Yes	135 (6.9%)	199 (11.8%)	259 (7.9%)	124 (5.6%)	602 (16.6%)	
Insurance	Insured	1,912 (97.8%)	1,644 (97.3%)	3,175 (97.3%)	2,164 (97.3%)	3,534 (97.5%)	0.831
	Uninsured	44 (2.2%)	45 (2.7%)	89 (2.7%)	61 (2.7%)	91 (2.5%)	
Location	Urban	1,944 (99.4%)	1,484 (87.9%)	2,521 (77.2%)	1,237 (55.6%)	2,680 (73.9%)	< 0.001
	Rural	12 (0.6%)	205 (12.1%)	743 (22.8%)	988 (44.4%)	945 (26.1%)	
Grade	I/II	730 (37.3%)	696 (41.2%)	1,247 (38.2%)	879 (39.5%)	1,452 (40.1%)	0.048
	III	1,146 (58.6%)	901 (53.3%)	1,872 (57.4%)	1,246 (56.0%)	2,024 (55.8%)	
	Unknown	80 (4.1%)	92 (5.4%)	145 (4.4%)	100 (4.5%)	149 (4.1%)	
Stage	I/II	1,616 (15.7%)	1,377 (13.3%)	2,638 (25.6%)	1,766 (17.1%)	2,926 (28.3%)	0.1063
	III	340 (14.0%)	312 (12.8%)	626 (25.7%)	459 (18.8%)	699 (28.7%)	
Lateral	Left	1,001 (51.2%)	851 (50.4%)	1,673 (51.3%)	1,166 (52.4%)	1,837 (50.7%)	0.710
	Right	955 (48.8%)	838 (49.6%)	1,591 (48.7%)	1,059 (47.6%)	1,788 (49.3%)	
Surgery	Yes	1,876 (95.9%)	1,599 (94.7%)	3,112 (95.3%)	2,136 (96.0%)	3,417 (94.3%)	0.008
	No	80 (4.1%)	89 (5.3%)	151 (4.6%)	85 (3.8%)	201 (5.5%)	
	Unknown		1 (0.1%)	1 (0.0%)	4 (0.2%)	7 (0.2%)	
Radiation	Yes	1,037 (53.0%)	810 (48.0%)	1,653 (50.6%)	1,116 (50.2%)	1,656 (45.7%)	< 0.001
	No	911 (46.6%)	869 (51.5%)	1,603 (49.1%)	1,092 (49.1%)	1,948 (53.7%)	
	Total	8 (0.4%)	10 (0.6%)	8 (0.2%)	17 (0.8%)	21 (0.6%)	
Chemo therapy	Yes	1,481 (75.7%)	1,237 (73.2%)	2,482 (76.0%)	1,659 (74.6%)	2,716 (74.9%)	0.244
	None/Unknown	475 (24.3%)	452 (26.8%)	782 (24.0%)	566 (25.4%)	909 (25.1%)	
Subtype	HER2+	1,956 (100.0%)	1,689 (100.0%)	3,264 (100.0%)	2,225 (100.0%)	3,625 (100.0%)	

### Kaplan-Meier survival estimates

**Fig. 2:**
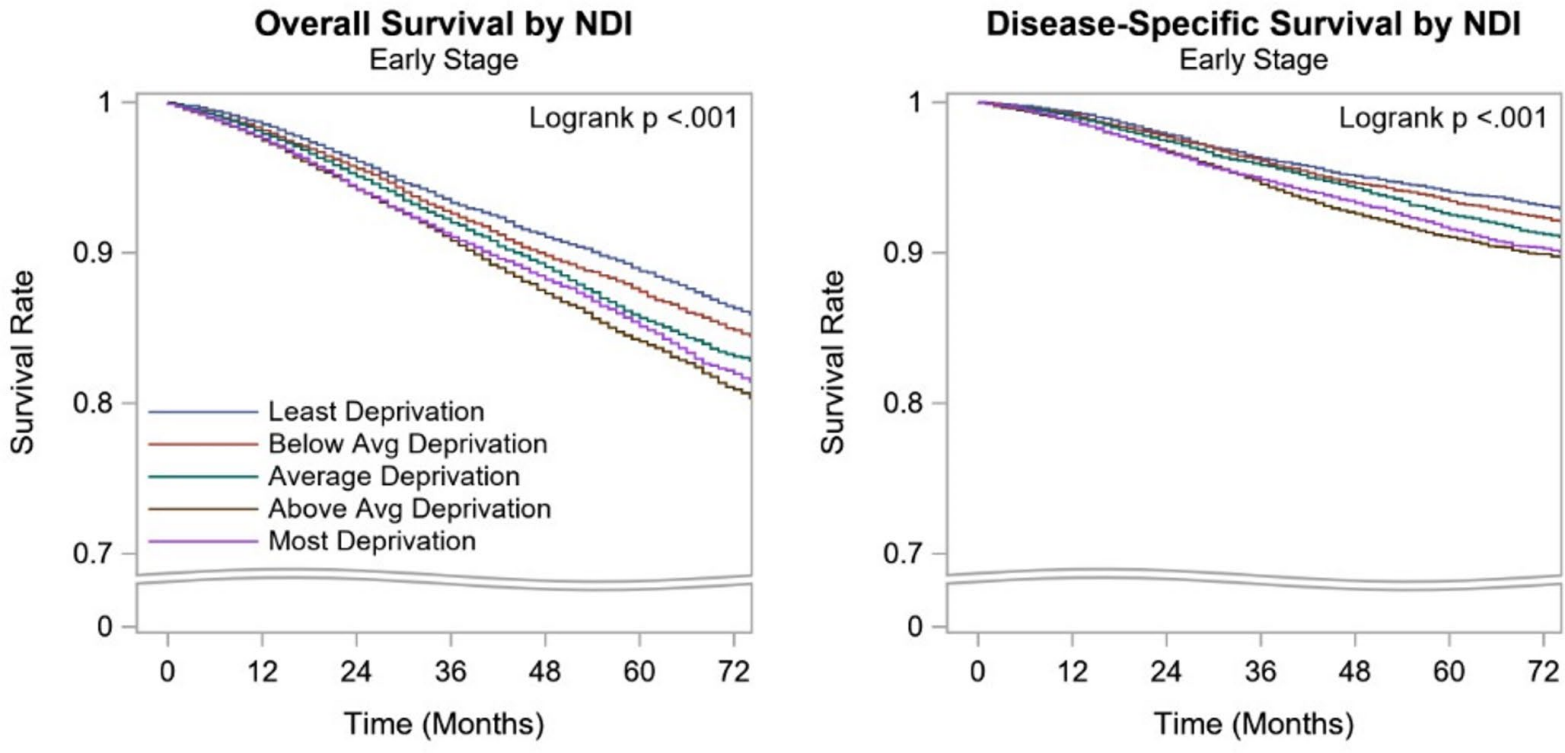
Kaplan-Meier Curves for overall and disease specific survival by NDI for locoregional breast cancer NDI: Neighborhood deprivation index, Avg: Average

On univariate analysis, after a median follow-up of 44 months, the 5-year OS rate of the overall cohort was 87%. The 5-year OS of the locoregional BC patients who live in the Q1 and Q2 quintile was lower when compared to those who live in the Q5 quintile (85%, 84% vs. 89%, p < 0.001). The DSS of the overall cohort also followed a similar pattern (DSS of Q1, Q2 vsQ5: 92%, 91% vs. 94%, p < 0.001) (Table 5; Fig. 2).

**Table 5:** Survival rates in locoregional overall cohort and breast cancer subtypes by NDI: overall survival and disease-specific survival

NDI Quintiles	5-yr Survival Rate (95% CI)	Median Follow-up (months) (Range)	Log Rank P-value
**Overall Cohort**			
**Overall Survival**			
Total	0.87 (0.87, 0.87)	44.0 (0.0, 83.0)	p < 0.001
Least Deprivation (Q5)	0.89 (0.88, 0.89)	54.0 (0.0, 83.0)	
Below Avg Deprivation (Q4)	0.87 (0.87, 0.88)	53.0 (0.0, 83.0)	
Average Deprivation (Q3)	0.86 (0.85, 0.86)	46.0 (0.0, 83.0)	
Above Avg Deprivation (Q2)	0.84 (0.83, 0.85)	49.0 (0.0, 83.0)	
Most Deprivation (Q1)	0.85 (0.85, 0.86)	49.0 (0.0, 83.0)	
**Disease-specific survival**			
Total	0.93 (0.93, 0.93)	42.0 (0.0, 83.0)	p < 0.001
Least Deprivation (Q5)	0.94 (0.94, 0.94)	52.0 (0.0, 83.0)	
Below Avg Deprivation (Q4)	0.93 (0.93, 0.94)	50.0 (0.0, 83.0)	
Average Deprivation (Q3)	0.92 (0.92, 0.93)	44.0 (0.0, 83.0)	
Above Avg Deprivation (Q2)	0.91 (0.90, 0.92)	46.0 (0.0, 83.0)	
Most Deprivation (Q1)	0.92 (0.91, 0.92)	46.0 (0.0, 83.0)	
**Triple-negative breast cancer**			
**Overall Survival**			
Total	0.76 (0.75, 0.77)	45.0 (0.0, 83.0)	p = 0.004
Least Deprivation (Q5)	0.78 (0.75, 0.80)	56.0 (0.0, 83.0)	
Below Avg Deprivation (Q4)	0.79 (0.76, 0.82)	53.0 (0.0, 83.0)	
Average Deprivation (Q3)	0.76 (0.73, 0.77)	48.0 (0.0, 83.0)	
Above Avg Deprivation (Q2)	0.74 (0.71, 0.76)	50.0 (0.0, 83.0)	
Most Deprivation (Q1)	0.75 (0.73, 0.76)	49.0 (0.0, 83.0)	
**Disease-specific survival**			
Total	0.81 (0.81, 0.82)	43.0 (0.0, 83.0)	p = 0.007
Least Deprivation (Q5)	0.83 (0.81, 0.86)	53.0 (0.0, 83.0)	
Below Avg Deprivation (Q4)	0.85 (0.82, 0.87)	50.0 (0.0, 83.0)	
Average Deprivation (Q3)	0.82 (0.80, 0.83)	46.0 (0.0, 83.0)	
Above Avg Deprivation (Q2)	0.80 (0.78, 0.82)	47.0 (0.0, 83.0)	
Most Deprivation (Q1)	0.81 (0.79, 0.82)	46.0 (0.0, 83.0)	
**Hormone-receptor positive breast cancer**			
**Overall Survival**			
Total	0.90 (0.89, 0.90)	43.0 (0.0, 83.0)	p < 0.001
Least Deprivation (Q5)	0.91 (0.90, 0.92)	52.0 (0.0, 83.0)	
Below Avg Deprivation (Q4)	0.90 (0.89, 0.90)	51.0 (0.0, 83.0)	
Average Deprivation (Q3)	0.88 (0.87, 0.89)	45.0 (0.0, 83.0)	
Above Avg Deprivation (Q2)	0.87 (0.87, 0.88)	48.0 (0.0, 83.0)	
Most Deprivation (Q1)	0.88 (0.88, 0.89)	47.0 (0.0, 83.0)	
**Disease-specific survival**			
Total	0.96 (0.95, 0.96)	42.0 (0.0, 83.0)	p < 0.001
Least Deprivation (Q5)	0.96 (0.96, 0.97)	50.0 (0.0, 83.0)	
Below Avg Deprivation (Q4)	0.96 (0.95, 0.96)	49.0 (0.0, 83.0)	
Average Deprivation (Q3)	0.95 (0.95, 0.96)	43.0 (0.0, 83.0)	
Above Avg Deprivation (Q2)	0.95 (0.94, 0.95)	46.0 (0.0, 83.0)	
Most Deprivation (Q1)	0.95 (0.94, 0.95)	45.0 (0.0, 83.0)	
**HER2- positive breast cancer**			
**Overall Survival**			
Total	0.87 (0.87, 0.88)	42.0 (0.0, 83.0)	p < 0.001
Least Deprivation (Q5)	0.89 (0.88, 0.91)	50.0 (0.0, 83.0)	
Below Avg Deprivation (Q4)	0.89 (0.87, 0.91)	49.0 (0.0, 83.0)	
Average Deprivation (Q3)	0.86 (0.84, 0.87)	43.0 (0.0, 83.0)	
Above Avg Deprivation (Q2)	0.84 (0.82, 0.86)	44.0 (0.0, 83.0)	
Most Deprivation (Q1)	0.85 (0.84, 0.87)	46.0 (0.0, 83.0)	
**Disease-specific survival**			
Total	0.92 (0.91, 0.92)	40.0 (0.0, 83.0)	p < 0.001
Least Deprivation (Q5)	0.93 (0.92, 0.94)	49.0 (0.0, 83.0)	
Below Avg Deprivation (Q4)	0.93 (0.91, 0.94)	47.0 (0.0, 83.0)	
Average Deprivation (Q3)	0.91 (0.89, 0.92)	42.0 (0.0, 83.0)	
Above Avg Deprivation (Q2)	0.89 (0.87, 0.91)	42.0 (0.0, 83.0)	
Most Deprivation (Q1)	0.90 (0.89, 0.91)	44.0 (0.0, 83.0)	

**Fig. 3:**
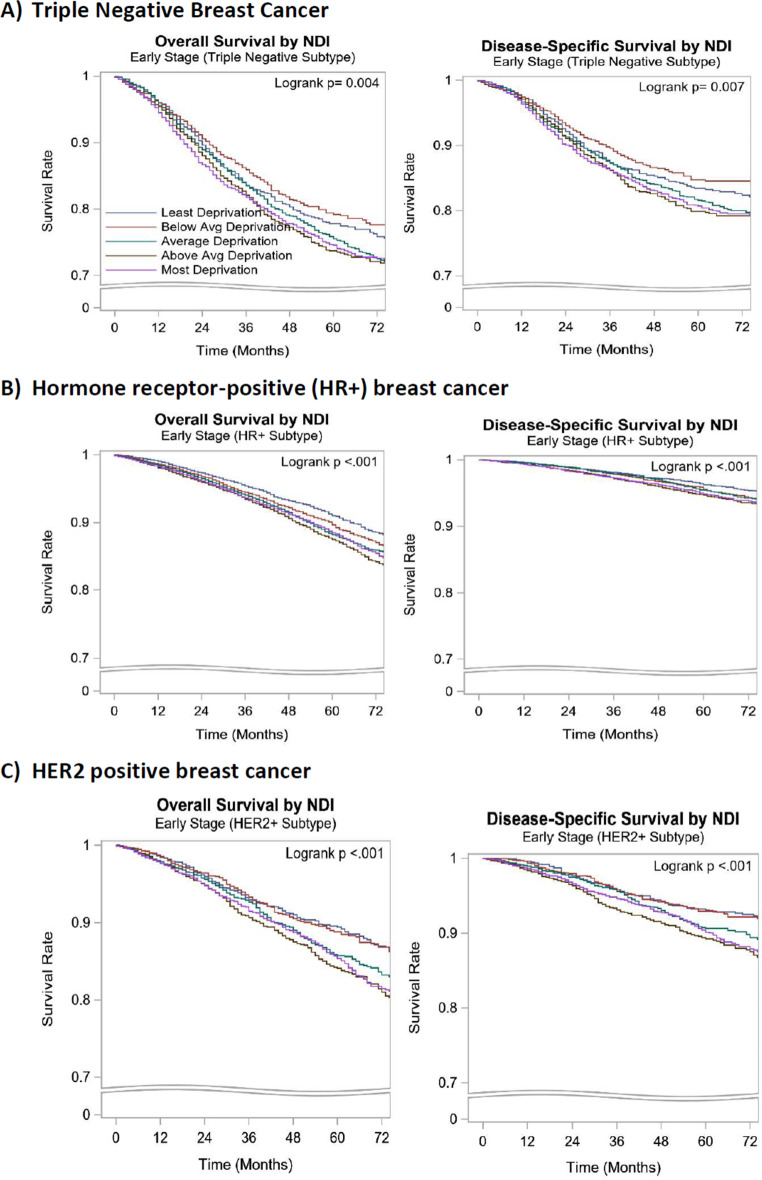
Kaplan-Meier Curves of overall and disease-specific survival by NDI for locoregional breast cancer subtypes NDI: Neighborhood deprivation index, Avg: Average, N: Number, HR+ : Hormone receptor-positive human epidermal growth factor 2- negative, HER2 + : Human epidermal growth factor receptor 2 positive

In subset analysis stratified by the BC subtypes, the 5-year OS and DSS were lower in the Q1 and Q2 quintiles compared to the Q5 quintile in all the subtypes of BC (HR+, HER2 + and TNBC). However, the 5-year DSS rate was not significantly different in the HR + subtype (Q1, Q2 vs. Q5: 95%, 95% vs. 96%, p < 0.001). (Table 5; Fig. 3).

### Multivariate survival analysis

**Fig. 4:**
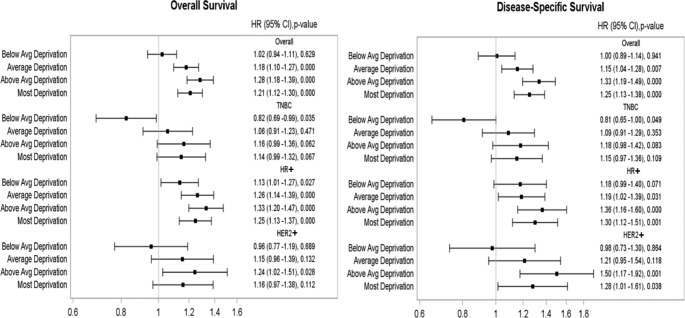
Multivariate survival analysis in the locoregional overall cohort: overall survival and disease-specific survival HR (95% CI): Hazard ratio (95% confidence interval), Avg: Average, TNBC: Triple negative breast cancer, HR+: Hormone receptor-positive human epidermal growth factor 2- negative, HER2+ : Human epidermal growth factor receptor 2 positive

In multivariate analysis after adjusting for socio-demographic, clinical, and treatment variables, in the overall cohort, those who live in Q2 quintile and Q1 quintile have inferior OS and DSS when compared to those who live in Q5 quintile (OS in Q2: Hazard Ratio (HR) 1.28; OS in Q1: HR 1.2; DSS in Q2: HR 1.33; DSS in Q1: HR 1.25, all p < 0.001). In the subset analysis, similar results for OS and DSS by NDI were observed in hormone receptor positive HER2 negative (HR+) and HER2 + subtypes, but not in TNBC (Fig. 4). In a separate multivariate cox regression model, age, race, insurance status, sub-type, disease grade, surgery, radiation, and chemotherapy were found to be independently associated with OS and DSS (Tables 6 and 7 respectively). The OS and DSS in locoregional BC by race are given in Table 8.

**Table 6:** Multivariate cox regression model of overall survival in the locoregional overall cohort

Overall Sample (OS) Early Stage		Hazard Ratio	95% CIs	P-value
NDI	Overall	1.054	1.037–1.071	< 0.0001
	Least Deprivation (Q5)	Ref.	Ref.	Ref.
	Below Avg Deprivation (Q4)	1.022	0.937–1.114	0.6289
	Average Deprivation (Q3)	1.180	1.095–1.271	< 0.0001
	Above Avg Deprivation (Q2)	1.282	1.184–1.387	< 0.0001
	Most Deprivation (Q1)	1.208	1.122–1.299	< 0.0001
Age	Continuous	1.052	1.050–1.054	< 0.0001
Race	White	Ref.	Ref.	Ref.
	Black	1.361	1.278–1.451	< 0.0001
	Other	0.753	0.664–0.854	< 0.0001
Hispanic	No	Ref.	Ref.	Ref.
	Yes	1.082	0.996–1.175	0.0623
Grade	I/II	Ref.	Ref.	Ref.
	III/IV	1.779	1.685–1.879	< 0.0001
	Unknown	1.313	1.172–1.472	< 0.0001
Insurance	Insured	Ref.	Ref.	Ref.
	Uninsured	1.591	1.373–1.844	< 0.0001
Surgery	Yes	Ref.	Ref.	Ref.
	No	3.726	3.461–4.010	< 0.0001
	Unknown	4.538	3.163–6.511	< 0.0001
Radiation	Yes	Ref.	Ref.	Ref.
	No	1.509	1.438–1.583	< 0.0001
	Total	1.111	0.760–1.626	0.5860
h Subtype	Triple Negative	Ref.	Ref.	Ref.
	HER2+	0.555	0.517–0.595	< 0.0001
	h+	0.524	0.492–0.558	< 0.0001
Chemotherapy	Yes	Ref.	Ref.	Ref.
	None / Unknown	0.887	0.839–0.939	< 0.0001

**Table 7:** Multivariate cox regression model of disease-specific survival in the locoregional overall cohort

Overall Sample (DSS) Early Stage		Hazard Ratio	95% CIs	P-value
NDI	Overall	1.068	1.045–1.092	< 0.0001
	Least Deprivation (Q5)	Ref	Ref	Ref
	Below Avg Deprivation (Q4)	1.005	0.889–1.136	0.9411
	Average Deprivation (Q3)	1.155	1.040–1.282	0.0070
	Above Avg Deprivation (Q2)	1.334	1.195–1.488	< 0.0001
	Most Deprivation (Q1)	1.251	1.130–1.385	< 0.0001
Age	Continuous	1.029	1.026–1.032	< 0.0001
Race	White	Ref.	Ref.	Ref.
	Black	1.348	1.241–1.464	< 0.0001
	Other	0.730	0.615–0.868	0.0003
Hispanic	No	Ref.	Ref.	Ref.
	Yes	1.137	0.992–1.264	0.0780
Grade	I/II	Ref.	Ref.	Ref.
	III/IV	2.610	2.412–2.825	< 0.0001
	Unknown	1.886	1.614–2.205	< 0.0001
Insurance	Insured	Ref.	Ref.	Ref.
	Uninsured	1.657	1.401–1.960	< 0.0001
Surgery	Yes	Ref.	Ref.	Ref.
	No	5.307	4.816–5.849	< 0.0001
	Unknown	5.494	3.489–8.653	< 0.0001
Radiation	Yes	Ref.	Ref.	Ref.
	No	1.252	1.171–1.338	< 0.0001
	Total	1.148	0.701–1.881	0.5830
h Subtype	Triple Negative	Ref.	Ref.	Ref.
	HER2+	0.502	0.460–0.548	< 0.0001
	h+	0.456	0.419–0.495	< 0.0001
Chemotherapy	Yes	Ref.	Ref.	Ref.
	None / Unknown	0.619	0.572–0.670	< 0.0001

**Table 8:** Multivariate analysis of the overall and disease-specific survival in the locoregional breast cancer by race

Overall Survival in the ‘White’ Racial Subgroup	Hazard Ratio	95% CIs	P-value	Disease-Specific Survival in the ‘White’ Racial Subgroup	Hazard Ratio	95% CIs	P-value
Least Deprivation (Q5)	Ref	Ref	Ref	Least Deprivation	Ref	Ref	Ref
Below Avg Deprivation (Q4)	1.048	0.953–1.153	0.33147	Below Avg Deprivation	1.039	0.907–1.191	0.57811
Average Deprivation (Q3)	1.185	1.092–1.286	0.00005	Average Deprivation	1.157	1.028–1.301	0.01539
Above Avg Deprivation (Q2)	1.321	1.210–1.442	< 0.001	Above Avg Deprivation	1.377	1.216–1.560	< 0.001
Most Deprivation (Q1)	1.236	1.139–1.340	< 0.001	Most Deprivation	1.291	1.151–1.448	< 0.001
Below Avg Deprivation (Q4)	0.805	0.617–1.048	0.10749	Below Avg Deprivation	0.757	0.537–1.069	0.11386
Average Deprivation (Q3)	1.183	0.962–1.456	0.11145	Average Deprivation	1.133	0.871–1.476	0.35211
Above Avg Deprivation (Q2)	1.178	0.958–1.448	0.11947	Above Avg Deprivation	1.225	0.942–1.591	0.12971
Most Deprivation (Q1)	1.202	0.989–1.462	0.06496	Most Deprivation	1.215	0.947–1.558	0.12623

## Discussion

Our study focuses on locoregional BC as its treatment requires access to health care systems that are less available in underprivileged neighborhoods. In our study, we found that the deprivation index of the neighborhoods was in significant association with BC survival. Our analysis showed that the OS and DSS of patients with locoregional BC were lower for those who live in socioeconomically underprivileged neighborhoods compared to those who live in affluent neighborhoods. The survival differences were observed among all subtypes of BC. The differences in survival persisted even after adjusting for demographic, clinical, and treatment factors that could affect BC survival. On multivariate analysis, the mortality difference among the patients living in different socioeconomic areas was statistically significant within the overall cohort, HR + and HER2 + BC subtypes, but not within the TNBC subtype. This could be explained by the aggressive nature of TNBC. As TNBC is very aggressive and has high relapse rate [18], the survival of patients with TNBC could be poor regardless of their socioeconomic status.

Understanding the impact of neighborhood deprivation on BC survival will facilitate the development and implementation of policies and prioritize investments in communities with high deprivation scores. This could improve the socioeconomic conditions which could eventually improve clinical outcomes [19]. Several factors in the neighborhood influence the health of an individual directly, as well as indirectly: poverty, access to the health care system, transportation system, housing quality, unemployment, environmental pollution including air and water pollution, neighborhood hygiene, waste management system, crime rates, racial composition, educational system, tobacco availability and marketing, access to healthy food [20–23]. These along with the factors that affect the individual such as marital status, family/social support, co-morbidities, mental health, nutritional status, healthy lifestyle, insurance status, and educational status play an inevitable role in the survival outcomes of malignancies. Studies have shown that prolonged and cumulative exposure to the above-mentioned deprivation-associated stressors can induce chronic inflammation which is one of the etiologies behind cancer development [24, 25]. Therefore, a proper understanding of the deprivation factors of an individual and their neighborhood is essential to plan interventions to reduce the burden of cancer mortality.

Our study adds to the existing literature in multiple ways. This study is the first to our knowledge to use a national database to examine the association between neighborhood deprivation with the clinical outcomes of locoregional BC; most prior studies used regional databases. Prior studies showed racial disparities in BC-related outcomes in the US and minoritized groups tend to have higher BC-related mortality [22]. In a study by Luningham et al. which examined the association between racial disparities and SES in BC survival between Black and White women across Georgia, it was found that Black women with BC had higher mortality than White women, but this disparity was not explained by the socioeconomic deprivation of their residential area. White patients living in socioeconomically affluent areas had lower rates of BC mortality compared to those who reside in underprivileged neighborhoods [26]. We observed similar results in our study: We observed similar results in our study. White BC patients living in socioeconomically underprivileged areas had higher mortality compared to Whites living in socioeconomically affluent areas. However, this difference in the mortality was not observed among Black BC patients. This is an interesting finding that may need validation in future studies. This difference leads us to speculate that irrespective of area of residence, Black breast cancer patients continue to have worse outcomes. This may be either due to cultural factors precluding Black patients to seek healthcare, mistrust for the system, lack of insurance, or could be due to inherent aggressive disease that even with access to the above facilities, the natural biology of the disease continues to be aggressive [27]. This clearly highlights the need for both better treatments for Black patients as well as more attention at a policy level for Black patients.

Our study finding critically shows the important role of area of residence on clinical outcomes, and thus emphasizing that socioeconomic factors of the neighborhood play a vital role in determining clinical outcomes. There were several studies conducted to understand the reason behind the observed racial disparities. One of them was attributed to poor neighborhoods. Black and Hispanic women are more likely to live in poor neighborhoods and Black patients were found to live in neighborhoods with high poverty rates and this difference persists even after adjusting for their income status [28]. In our study, we found that Black and Hispanic women with BC were more commonly residing in the underprivileged neighborhoods compared to socioeconomically affluent neighborhoods; however, the disparities in BC-related mortality of the patients from these socioeconomically different neighborhoods was not observed among Black patients and persisted even after accounting for the racial disparities.

Similarly, patients without insurance were found more commonly in the underprivileged neighborhoods and those neighborhoods had more rural areas. Advanced BCs (higher stage and grade) and aggressive BC such as HER2 + and TNBC were predominantly found in the underprivileged neighborhoods compared to the affluent neighborhoods. In our study, BC patients from affluent neighborhoods received more surgical and radiation treatments which can be explained by the higher percentages of urban areas in these regions with better facilities for treatments, and better referral systems. In a study by Fwelo et al., it was found that Black and Hispanic women were more likely to undergo mastectomy compared to Whites [29]. This discrepancy could be attributed to the fact that breast cancer is often diagnosed when locally advanced in Black women, indicating limited access to early detection through adequate screening and precluding lumpectomy [27, 29]. Additionally, cultural preferences, low healthcare literacy, and transportation barriers may contribute to limited healthcare access, potentially influencing the decision to avoid radiation treatment [27, 30]. Our study showed that in the overall BC cohort, patients who received chemotherapy were slightly higher in the underprivileged neighborhoods than in the affluent neighborhood. One possible explanation for this is that the advanced diseases that requires chemotherapy was more prevalent in the underprivileged neighborhood regions. This could also be attributed to several other factors, such as low healthcare education, leading to misconceptions about medical information, as well as limited access to tests (due to insurance issues) that predict the benefits of adjuvant chemotherapy, like the Oncotype DX 21-gene expression assay, which can potentially result in overtreatment among racial minorities [27, 31]. Nevertheless, the disparities in BC-related mortality remain unaffected when adjusted for the demographic, clinical, pathological, and treatment-related factors such as age, race, stage, grade, insurance, surgery, radiation, and chemotherapy. This suggests that additional factors related to neighborhood SES that are not captured by the NDI play an important role in BC-related outcomes. The access to genetic and somatic testing which are important for deciding the appropriate treatments in BC might be limited to patients from poor neighborhood which could have impacted their survival.

Poverty, unhealthy food habits, decreased access to healthy food, environmental pollution, increased advertising of tobacco in poor neighborhood leads to increased incidence of various cancers in patients from these neighborhoods [20, 21, 23, 32, 33]. Furthermore, the transportation barriers, decreased access to better comprehensive cancer centers with standard of care treatments or novel clinical trials, poor nutritional and educational status of patients, financial toxicity associated with cancer treatment leads to increased cancer-related mortality in socioeconomically poor neighborhoods [11, 15, 34, 35]. In addition to this, poor environmental hygiene and pollution can add to the increased mortality by causing infections in cancer patients who are already immunocompromised due to cancer and associated treatments [36]. In a patient-reported outcomes study in advanced cancers, it was found that patients from socioeconomically underprivileged areas have a higher level of anxiety [37]. Factors such as anxiety, depression, and poor social support which are subjective measures of poor mental health are not accounted for in any of the tools to measure the neighborhood/individual SES and have been shown to be associated with cancer-related mortality [14, 37, 38]. Disparities in BC survival related to neighborhood SES reflect the systematic barriers in policies related to health care, education, employment, insurance, environment, and judicial system. Our study findings may support restructuring the policies, to implement new policies and investments in socioeconomically underprivileged neighborhoods which would help to decrease inequalities in opportunities, improve healthcare facilities, and increase access to timely cancer treatments.

Our study has many strengths and certain limitations. We used large real-world data to assess the impact of neighborhood deprivation on clinical outcomes of BC patients. These data capture more than 50% of the US population, and therefore, the results are generalizable. We adjusted for multiple factors that are known to influence survival, including racial distribution, demographic factors, clinicopathological factors of the disease [39]. Although NDI is a comprehensive tool to assess socioeconomic disadvantage, it may not capture all the factors associated with neighborhood SES. We could not assess the influence of several neighborhood factors that may contribute to the mortality of BC such as access to transportation, environmental: air, water pollution, poverty level, accessibility to healthy food, and crime rate of the neighborhood. As we do not have one comprehensive tool to assess the socioeconomic status of neighborhoods and individuals together, future studies warranting the combination of multiple indices such as area deprivation index, Yost index might be beneficial. As it is a retrospective national database study, several individual factors that can affect the mortality rates of BC such as comorbidities of patients, social support, details of factors such as anxiety, and depression that can affect the mental and physical condition of the patients were not collected. Incompleteness of individual-level data collected on cancer risk and treatment, and incomplete values for several variables collected from multiple registries were other limitations. Further tumor recurrence data, specific details on the type, dose, and duration of chemotherapy, radiation, oral chemotherapy, targeted agents were not available in the SEER database and these factors could have been associated with the mortality of BC.

Moving forward, it is crucial for professional societies and cancer institutes to take the initiative to design strategies that address breast cancer disparities stemming from the socioeconomic status of neighborhoods. Some potential initiatives include identifying underprivileged areas in communities and providing additional facilities to these patients, such as offering free transportation and, organizing mobile units equipped with imaging technologies to visit underprivileged areas regularly. Additionally, addressing the climate gap through federal support to rebuild infrastructure, reduce sources of pollution, and ensure accessible and affordable clean energy sources can be another strategy to alleviate economic injustice [40]. Restructuring the health system to address the racial wealth gap, promoting equity and inclusion by ensuring adequate access to clinical trials for everyone, supporting research focused on preventing and treating aggressive breast cancer subtypes (more prevalent in racial minorities, such as Blacks), and ensuring universal access to healthcare, are key strategies that can be adopted to decrease healthcare disparities and reduce mortality rates from breast cancer [5, 41, 42].

## Conclusion

The findings from our study suggest that neighborhood deprivation is significantly and independently associated with worse clinical outcomes among patients with BC in the US. Locoregional BC patients from areas with worse NDI have poor OS and DSS, after accounting for demographic, clinicopathological, and treatment-related factors. Identification of these poor-resource neighborhoods is critical to guide investments in these neighborhoods and implement policies focusing on improving the SES of these areas with high deprivation to reduce healthcare disparities and improve breast cancer outcomes. Future studies are warranted to understand the factors affecting the neighborhood socioeconomic status other than what is mentioned in our study and to assess their relationship with BC-related survival. The data from these studies might be extrapolated to other cancers which would help us to improve the quality of life of patients and cancer-related mortalities.

## Data Availability

All data utilized in this article is available in public datasets such as SEER and NCI Neighborhood deprivation index. Data analyzed during this study are included in this published article and its Appendix.
